# Impact of stearic acid and oleic acid on hemostatic factors in the context of controlled diets consumed by healthy men

**DOI:** 10.1038/ejcn.2014.62

**Published:** 2014-04-16

**Authors:** S K Gebauer, R P Tracy, D J Baer

**Affiliations:** 1 USDA, Agricultural Research Service, Beltsville Human Nutrition Research Center, Beltsville, MD, USA; 2 Department of Pathology, University of Vermont College of Medicine, Colchester, VT, USA

## Abstract

The effects of stearic acid (STA) on cardiovascular disease risk beyond lipid and lipoprotein risk factors, including hemostasis, are unclear, particularly when compared with unsaturated fatty acids. The aim of the present study is to compare the effects of STA with those of oleic acid (OL) on markers of hemostasis. In a randomized crossover study, 50 men consumed six controlled diets for 5 weeks each (39% energy from fat, 15% energy from protein, 46% energy from carbohydrate (CHO)). Fat (8% energy) was replaced across diets by: STA, OL, CHO (control), *trans* fatty acids (TFAs), TFA/STA and 12:0–16:0 saturated fatty acids. Factor VIIc, plasminogen activator inhibitor-1 (PAI-1) and plasmin alpha-2-antiplasmin complex concentrations were not different between OL and STA (*P*>0.05). Compared with control, OL increased factor VIIc and PAI-1 (*P*⩽0.05), whereas there were no differences with STA (*P*>0.05). STA and OL similarly affect markers of hemostasis in healthy men, within the context of a highly controlled diet.

## Introduction

Over the past decade, dietary guidance and regulations have aimed to limit *trans* fatty acid (TFA) and saturated fatty acid (SFA) intake and the amount in the food supply, which has resulted in the need for TFA alternatives that are practical and safe. Stearic acid (STA) may serve as a viable alternative to TFAs, particularly in foods that require solid fats (such as margarines, shortenings and baked goods), owing to physical characteristics (solid at room temperature) as well as neutral or beneficial cholesterolemic effects compared with other SFAs and TFAs.^1,2^ Trait-enhanced oils that are higher in oleic acid (OL) may be a practical alternative for frying applications; monounsaturated fatty acids have been shown to have a beneficial impact on blood lipids compared with SFAs and TFAs. Although studies have compared STA with other SFAs,^2^ data are lacking from highly controlled feeding studies that compare the effects of STA with unsaturated fatty acids, particularly on markers of cardiovascular risk beyond traditional lipoprotein risk factors. Recognizing the lack of data, the 2010 Report of the Dietary Guidelines Advisory Committee specifically addresses the need for more research on other metabolic effects of STA, including coagulation, particularly when replaced for unsaturated fatty acids in the diet. We have previously published the effects of fatty acids on lipids^3^ and markers of inflammation^4^ in the context of a controlled diet. Expanding on these findings, we now report on the effects of STA and OL on markers of coagulation and fibrinolysis.

## Subjects and methods

Details of study design, methods and baseline characteristics have been previously reported in detail.^3,4^ Briefly, men were recruited between the ages of 25 and 60 years, and determined by a physician to be in good health. Inclusion criteria were as follows: high-density lipoprotein-cholesterol >0.65 mmol/l, triacylglycerol <3.39 mmol/l and within 85–120% of their gender-specific ideal body mass index. Participants who were taking lipid-lowering or blood pressure-lowering medications, dietary supplements or who had eating habits inconsistent with the study protocol were excluded. All procedures were in accordance with the ethical standards of the Declaration of Helsinki and were approved by the Johns Hopkins University Committee on Human Research.

Participants were instructed to eat all foods and only foods provided to them by the Beltsville Human Nutrition Research Center. Diets were planned to vary by 8% energy as follows: (1) carbohydrate (CHO) diet (control): 8.5% energy from fat replaced by digestible carbohydrate; (2) OL diet: 8% energy from oleic acid; (3) LMP diet: 8% energy from SFAs, as the sum of lauric (L), myristic (M) and palmitic acids (P); (4) STA diet: 8% energy from STA; (5) TFA diet: 8% energy from TFAs; and (6) TFA/STA diet: 4% energy from TFAs and 4% energy from STA (Table 1). Details regarding the fat sources and composition have been previously reported.^3,4^

Blood was collected following an overnight fast. Plasma was separated by centrifugation at 1400 *g* for 20 min at 4 °C and was aliquotted into cryovials. All samples were stored at −80 °C until the end of the intervention, at which point all analyses were conducted. Factor VIIc was measured by a clot-rate assay with Stago ST4 instrumentation, according to Cushman *et al.*,^5^ using human factor VII-immunodeficient plasma (Baxter-Dade, Bedford, MA, USA) and Thromborel S human placenta-derived thromboplastin (Behring Diagnostics, Marburg, Germany). The plasminogen activator inhibitor-1 (PAI-1) assay was based on the method of DeClerck *et al.*,^6^ which is sensitive to free PAI-1 but not to PAI-1 in a complex with tissue-type plasminogen activator. Plasmin alpha-2-antiplasmin complex (PAP) was measured by an assay that detects only complex and not free plasmin or antiplasmin.^7^

All analyses were performed with SAS (Cary, NC, USA) or S-Plus, using a mixed-effects model for analysis of the data. Data were analyzed by using an analysis of variance model that included terms for diet, period, subject and carryover effect. *A priori*, it was established that for each significant effect of diet, a Tukey–Kramer test would be used to determine differences among treatments. Differences were considered significant at *P*⩽0.05. Data for PAI-1 were log transformed to correct for problems of heterogeneity of variance and are presented as log transformed.

## Results

There were no differences in plasma concentrations of factor VIIc when comparing the STA diet with the OL diet. Furthermore, there were no differences in factor VIIc among the STA, TFAs, TFA/STA and CHO (control) diets (Table 2).

Plasma concentrations of PAP were not significantly different when comparing the STA diet with the other treatment diets. The LMP diet increased PAP compared with the CHO diet, whereas all other treatment diets were not different from the CHO diet.

There were no differences in plasma concentration of PAI-1 when comparing the STA diet with the OL diet. Compared with the CHO diet, PAI-1 level increased following the OL diet, and remained unchanged following the other treatment diets.

## Discussion

The effects of STA on cardiovascular disease risk beyond lipid and lipoprotein risk factors, including hemostasis, are unclear, particularly when compared with unsaturated fatty acids. Factor VII has a major role in thrombus formation, whereas PAI-1 is the major regulator of fibrinolysis. PAP is a marker of activation of the fibrinolytic system. Plasma concentrations of factor VIIc, PAI-1 and PAP have been associated with increased risk of cardiovascular disease and coronary events.^8,9^ In the present study, STA and OL had a similar impact on the plasma concentration of factor VIIc, PAI-1 and PAP. When compared with the CHO diet, OL increased PAI-1 and factor VIIc, whereas STA did not. When compared with LMP, STA decreased factor VIIc, which is consistent with the literature.^2^ In another controlled study in healthy adults (*n*=45), there were no differences in factor VII, PAI activity and tissue plasminogen activator/PAI-1 complex when comparing STA, OL and linoleic acid (~7% of energy each).^10^ STA and OL also had a similar impact on factor VII in the postprandial state.^2^

In the present study, the high-STA test fat contained 43.9% of fatty acids as STA, which is similar to amounts contained in fats that are naturally rich in STA, such as sheanut oil (39%) and cocoa butter (34%). If used as an alternative to TFAs, estimates indicate that STA intake would be ~3.7–4.5% of energy.^2^ Many studies, including the present study, have investigated the effects of STA at much higher amounts. Overall, data from controlled studies suggest that intake of STA, in amounts as high as 9% of energy and potentially up to 14% of energy, results in little or no adverse effect on hemostatic risk factors. Even at consumption of these high amounts (⩾10% of energy), there was no evidence of adverse effects of STA on the hemostatic factors reported herein. Furthermore, STA and OL had comparable effects on markers of hemostasis. A limitation of this study is the difficulty in establishing the biological significance of the changes in these markers within the broader context of cardiovascular disease risk. Strengths of this study are the tightly controlled diets and large sample size. In conclusion, these data on markers of hemostasis, along with previously published results on lipids and inflammation,^3,4^ demonstrate that STA is a suitable and perhaps more healthful replacement for TFAs in food applications that require a solid fat.

## Figures and Tables

**Table 1:** Fatty acid composition of diets

	*Diets*					
	*STA*	*OL*	*TFA*	*TFA/STA*	*CHO*	*LMP*
Total fat	138±2.6 (39.9)	133±2.3 (38.2)	137±2.6 (39.4)	136±2.6 (39.6)	105±2.0 (30.5)	138±2.6 (39.7)
Sum LMP	34.7±0.66 (10.0)	33.7±0.60 (9.7)	35.0±0.65 (10.1)	34.4±0.65 (10.0)	33.6±0.64 (9.8)	62.6±1.18 (18.0)
STA	37.8±0.72 (10.9)	10.1±0.18 (2.9)	9.7±0.18 (2.8)	23.8±0.45 (6.9)	10.0±0.19 (2.9)	9.4±0.18 (2.7)
OL (*cis* 18:1)	36.4±0.69 (10.5)	61.2±1.08 (17.6)	36.7±0.69 (10.6)	36.5±0.68 (10.6)	36.0±0.68 (10.5)	36.5±0.69 (10.5)
*trans* 18:1	1.0±0.02 (0.3)	0.3±0.01 (0.1)	28.8±0.54 (8.3)	14.5±0.27 (4.2)	0.7±0.01 (0.2)	0.7±0.01 (0.2)
Linoleic acid	15.3±0.29 (4.4)	13.2±0.23 (3.8)	13.9±0.26 (4.0)	14.8±0.28 (4.3)	13.0±0.25 (3.8)	14.6±0.28 (4.2)

Abbreviations: CHO, carbohydrate (control); LMP, lauric, myristic, palmitic acids; OL, oleic acid; STA, stearic acid; TFA, trans fatty acid.

All values are expressed as mean±s.e.m. in g/day (% of energy is expressed in parentheses). Detailed nutrient composition of diets has been previously reported.^3,4^

**Table 2:** Concentrations of plasma markers of hemostasis (*n*=50, mean and 95% confidence interval in parenthesis)^1^

	*Diets*					
	*STA*	*OL*	*TFA*	*TFA/STA*	*CHO*	*LMP*
Factor VIIc (%)	104^a,b^ (100, 108)	105^b,c^ (101, 109)	103^a,b^ (99, 105)	104^a,b^ (100, 108)	101^a^ (97, 105)	108^c^ (104, 110)
PAI-1 ln(ng/ml)	2.82^a,b^ (2.60, 3.04)	2.95^b^ (2.73, 3.17)	2.91^a,b^ (2.69, 3.02)	2.79^a^ (2.57, 3.01)	2.80^a^ (2.58, 3.02)	2.89^a,b^ (2.67, 3.11)
PAP (nM)	5.2^a,b^ (4.8, 5.6)	5.0^a^ (4.6, 5.4)	5.3^a,b^ (4.9, 5.5)	5.1^a,b^ (4.7, 5.5)	5.0^a^ (4.6, 5.4)	5.4^b^ (5.0, 5.6)

Abbreviations: CHO, carbohydrate (control); LMP, lauric, myristic, palmitic acids; OL, oleic acid; PAI-1, plasminogen activator inhibitor-1; PAP, plasmin alpha-2-antiplasmin; STA, stearic acid; TFAs, trans fatty acids.

1 Means within a row that do not share a common superscript letter are significantly different at *P*⩽0.05.
